# Development of a hydroxamamide-based bifunctional chelating agent to prepare technetium-99m-labeled bivalent ligand probes

**DOI:** 10.1038/s41598-021-98235-x

**Published:** 2021-09-21

**Authors:** Yoichi Shimizu, Masato Ando, Shimpei Iikuni, Hiroyuki Watanabe, Masahiro Ono

**Affiliations:** 1 Department of Patho-Functional Bioanalysis, Graduate School of Pharmaceutical Sciences, Kyoto University, 46-29, Yoshida Shimoadachi-cho, Sakyo-ku, Kyoto, 606-8501 Japan; 2 Department of Diagnostic Imaging and Nuclear Medicine, Graduate School of Medicine, Kyoto University, 54 Shogoin-kawahara-cho, Sakyo-ku, Kyoto, 606-8507 Japan

**Keywords:** Drug discovery, Oncology

## Abstract

Hydroxamamide (Ham) is a thiol-free chelating agent that forms technetium-99m (^99m^Tc)-complexes with a metal-to-ligand ratio of 1:2 under moderate reaction conditions. Therefore, Ham-based chelating agents will produce ^99m^Tc-labeled compounds with a bivalent targeting scaffold. For their universal usage, we developed a novel Ham-based bifunctional chelating agent, “Ham-Mal”, with a maleimide group that can easily conjugate with a thiol group, for to preparing ^99m^Tc-labeled bivalent ligand probes. Ham-Mal was synthesized by a four-step reaction, and then reacted with cysteine or c(RGDfC) to produce Ham-Cys or Ham-RGD. These precursors were reacted with ^99m^TcO_4_^-^ for 10 min under room temperature to obtain ^99m^Tc-(Ham-Cys)_2_ and ^99m^Tc -(Ham-RGD)_2_. The cellular uptake level of ^99m^Tc-(Ham-RGD)_2_ by U87MG (high Integrin ɑ_v_β_3_ expression) cells was significantly higher than that by PC3 (low Integrin ɑ_v_β_3_ expression) cells at 60 min after the incubation, and the uptake was significantly suppressed by pre-treatment for 15 min with excess c(RGDfK) peptide. In the in vivo study with U87MG/PC3 dual xenografted BALB/c-nu mice, the radioactivity of U87MG tumor tissue was significantly higher than that of PC3 tumor tissue at 360 min after the administration of ^99m^Tc-(Ham-RGD)_2_. These results suggest Ham-Mal may have potential as a bifunctional chelating agent for ^99m^Tc-labeled bivalent ligand probes.

## Introduction

Technetium-99m (^99m^Tc) is one of the major radioisotopes widely used for clinical single-photon emission computed tomography (SPECT) imaging because it emits gamma rays with a photon energy of 141-keV, which is suitable for detection by the SPECT scanner, and it is easily produced by a molybdenum-99/technetium-99m generator^1^. To radiolabel ^99m^Tc, many chelating moieties have been developed and used widely^2–4^.

Hydroxamamide (Ham) is a thiol-free chelating agent of oxo-technetium(V) [Tc(V)] known to produce ^99m^Tc-complexes with a high radiochemical yield under moderate reaction conditions^5–8^. In addition, Ham makes it possible to easily produce ^99m^Tc-labeled compounds with a bivalent targeting scaffold because it forms ^99m^Tc-complexes with a metal-to-ligand ratio of 1:2^7^. Our group previously developed Ham-based ^99m^Tc radiolabeled SPECT imaging probes and demonstrated their properties for detecting their target biomolecules^9–13^. In these probes, the Ham group was directly incorporated into their chemical structures, that is to say, the reaction for induction of Ham group was not independent on the synthesis of those probes in most cases. Therefore, to develop novel Ham-based ^99m^Tc radiolabeled probes with other targeting ligands, synthetic routes for the probes must be redesigned and established, which makes them difficult to produce. Bifunctional chelating agents composed of a metal binding moiety and a chemically reactive functional group simplify the introduction of chelating moieties to the targeting ligands^14^. However, no Ham-based bifunctional chelating agent for producing ^99m^Tc-labeled bivalent ligand probes has been reported.

**Figure 1:**
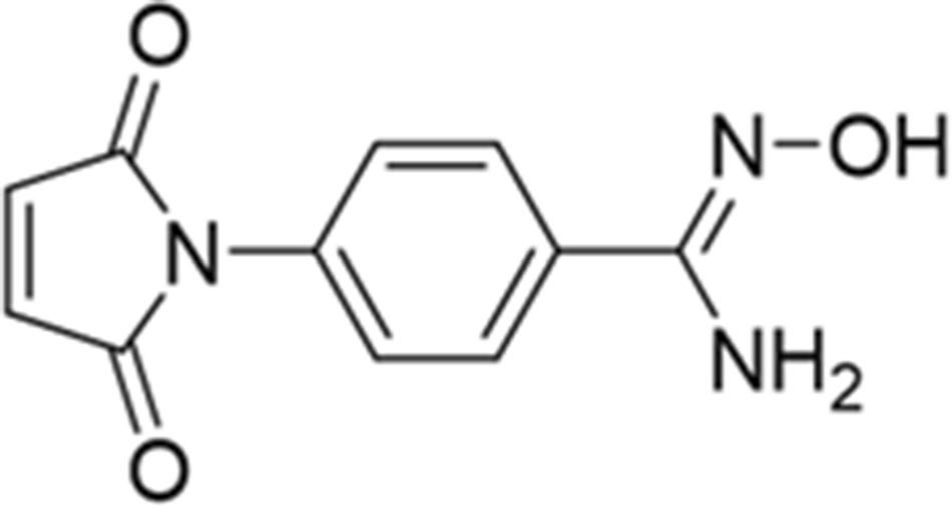
Chemical structure of Ham-Mal.

In this study, we designed and synthesized a novel Ham-based bifunctional chelating agent “Ham-Mal” to simplify the development of Ham-based SPECT imaging probes (Fig. 1). As a chemically reactive functional group, we chose the maleimide group, which can be easily reacted with thiol groups of ligands via Thio-Michael addition under moderate conditions^15^. To estimate the conditions of the ^99m^Tc radiolabeling reaction, we synthesized “Ham-Cys”, which was acquired by the reaction with Ham-Mal and cysteine, and then performed the ^99m^Tc radiolabeling reaction to produce “^99m^Tc-(Ham-Cys)_2_” under several reaction conditions. In addition, to evaluate the utility of Ham-Mal as a ^99m^Tc-bifunctional chelating agent, we synthesized “^99m^Tc-(Ham-RGD)_2_” whose precursor, “Ham-RGD”, was acquired by reacting Ham-Mal with c(RGDfC), a cyclic arginine-glycine-aspartic acid (RGD) peptide with a high affinity to Integrin ɑ_v_β_3_, a member of the integrin superfamily of adhesion molecules^16^.

## Results

### Chemistry

**Figure 2:**
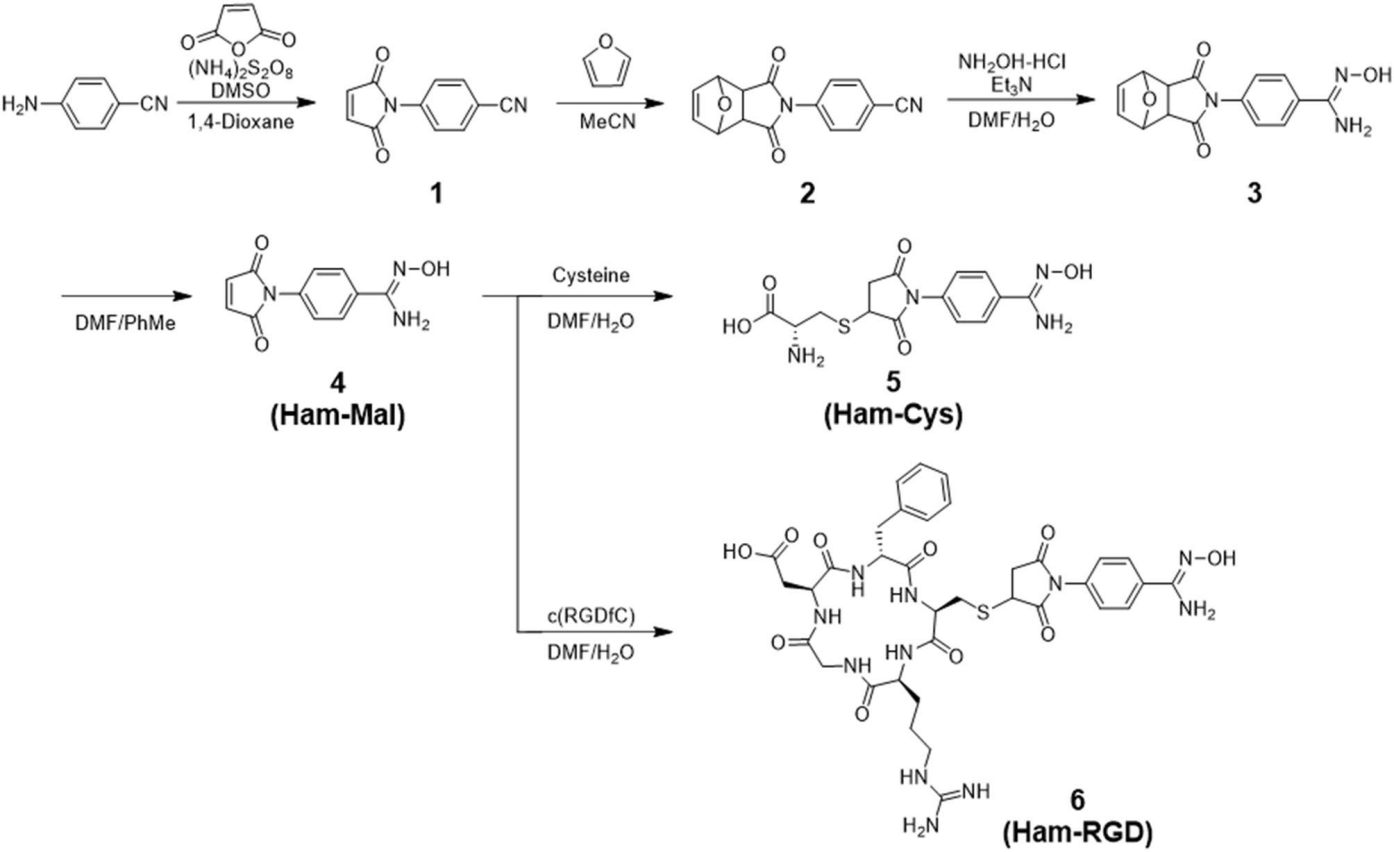
Synthetic route of Ham-Mal, Ham-Cys, and Ham-RGD.

The synthesis of Ham-Mal, Ham-Cys, and Ham-RGD is outlined in Fig. 2. Ham-Mal was prepared in four steps from 4-aminobenzonitrile. First, 4-aminobenzonitrile was reacted with maleic anhydride to acquire **1** and then its maleimide group was protected by furan to produce **2**. A Ham group was then introduced into **2** by reacting with hydroxylamine to produce **3**. Next, Ham-Mal (**4**) was acquired by deprotecting the maleimide group of **3**, with a total yield of 7.2% from 4-aminobenzonitrile. Ham-Cys and Ham-RGD were acquired by reacting Ham-Mal (**4**) with cysteine and c(RGDfC), with yields of 23% and 64%, respectively.

### Radiolabeling

**Figure 3:**
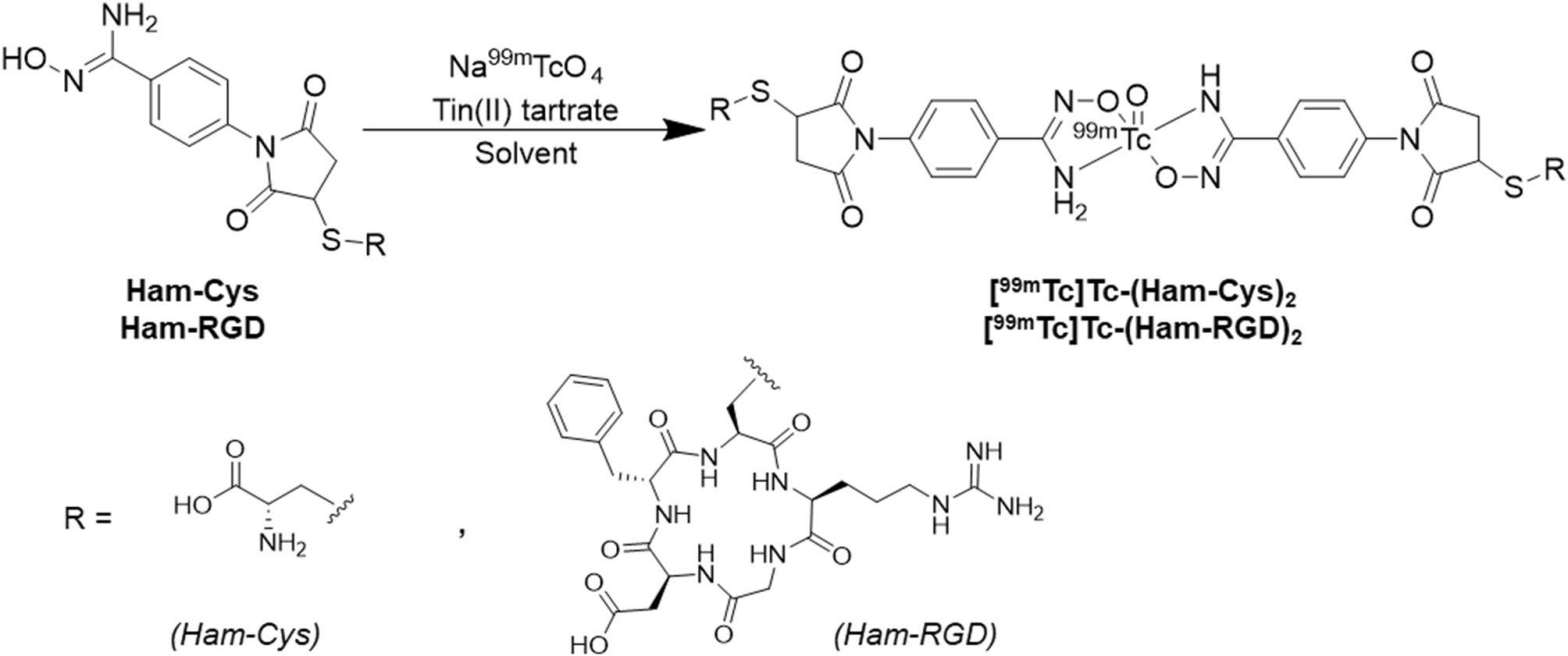
^99m^Tc labeling route of ^99m^Tc-(Ham-Cys)_2_ and ^99m^Tc-(Ham-RGD)_2_.

**Figure 4:**
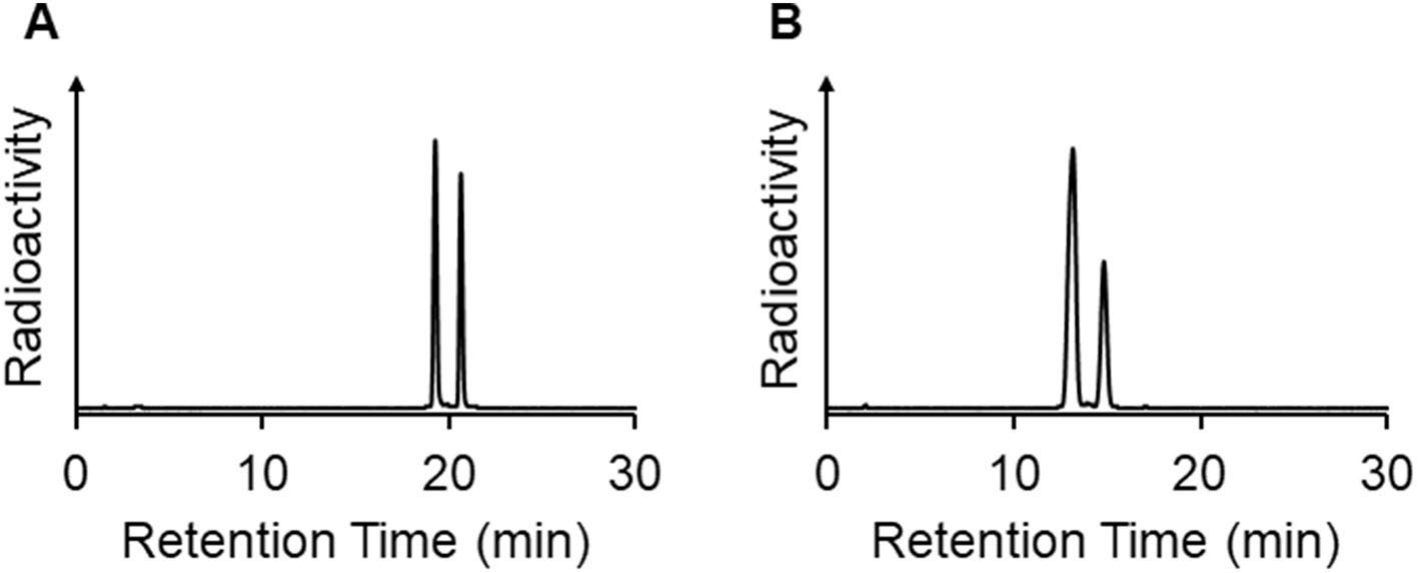
Radiochromatograms for ^99m^Tc-(Ham-Cys)_2_ **(A)** and ^99m^Tc-(Ham-RGD)_2_ **(B)**.

The ^99m^Tc labeling of Ham-Cys was performed by reacting it with ^99m^Tc pertechnetate and tin(II) tartrate hydrate as a reducing agent in a solvent of acetic acid/ethanol (1/4) or phosphate buffer (pH 7.4), with different final concentrations of Ham-Cys (10 or 20 mM) (Fig. 3). The radiochemical yields of ^99m^Tc-(Ham-Cys)_2_ reacted in phosphate buffer were markedly higher than those in acetic acid/ethanol solvent [phosphate buffer: 68.7 ± 2.4% (final concentration of Ham-Cys: 10 mM), 75.3 ± 4.5% (20 mM), acetic acid/ethanol solvent: 10.4 ± 9.8% (10 mM), 11.0 ± 5.0% (20 mM)] (Table 1). On the other hand, the concentration of the precursor (Ham-Cys) did not affect the radiochemical yield (Table 1). ^99m^Tc-(Ham-RGD)_2_ was obtained by reacting Ham-RGD (final concentration: 10 mM) with ^99m^Tc pertechnetate and tin(II) tartrate hydrate as a reducing agent in the solvent of phosphate buffer (pH 7.4), with a radiochemical yield of a 49.0 ± 4.8% and radiochemical purity of over 95% (Fig. 4). We also confirmed the purified ^99m^Tc-(Ham-Cys)_2_ and ^99m^Tc-(Ham-RGD)_2_ did not contained the respective precursor (Ham-Cys and Ham-RGD, respectively) (Supplementary file, Figure S1).

**Table 1:** Radiochemical yields of ^99m^Tc-(Ham-Cys)_2_ under each condition.

Solvent	Final concentration of Ham-Cys (mM)	Radiochemical yield (%)
Phosphate buffer (pH 7.4, 10 mM)	10	68.7 ± 2.4
	20	75.3 ± 4.5
Acetic acid/ethanol (1/4)	10	10.4 ± 9.8
	20	11.0 ± 5.0

Each value is the mean ± standard error of three independent experiments.

### In vitro cellular uptake study

**Figure 5:**
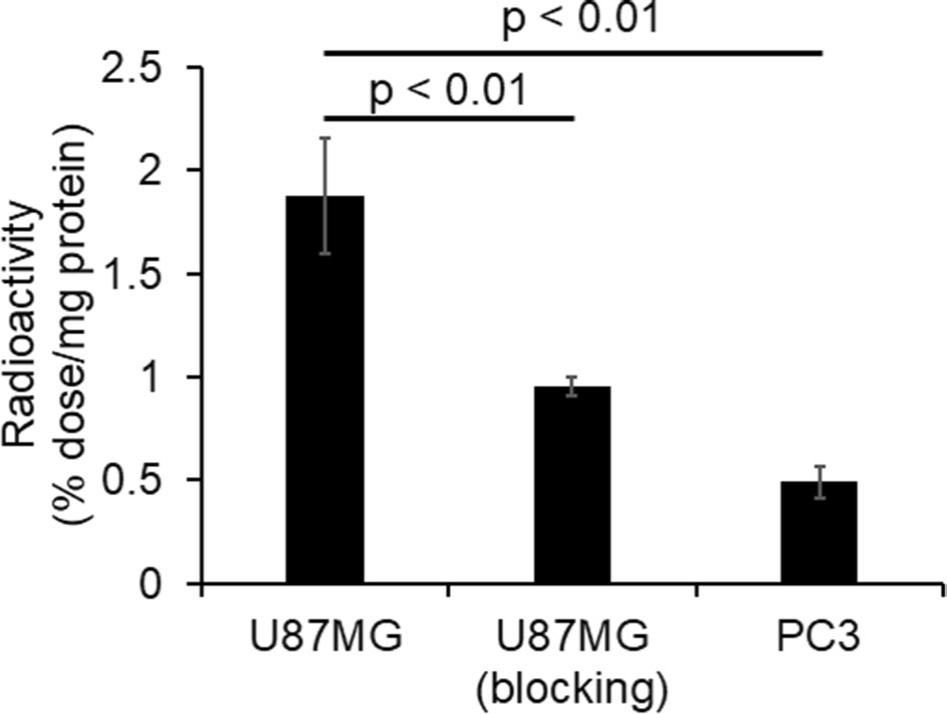
In vitro cellular binding of ^99m^Tc-(Ham-RGD)_2_ in U87MG and PC3 cells. Results are expressed as the mean ± standard error of six independent experiments.

The cellular uptake of ^99m^Tc-(Ham-RGD)_2_ is expressed as the %dose/mg protein (Fig. 5). The radioactivity of U87MG cells was significantly higher than that of PC3 cells (U87MG: 1.87 ± 0.28 vs. PC3: 0.49 ± 0.08%dose/mg protein, p < 0.01). In addition, the radioactivity of U87MG cells was significantly reduced by pretreatment with excess c(RGDfK) (0.95 ± 0.04%dose/mg protein, p < 0.01) (Fig. 5).

### In vitro stability of ^99m^Tc-(Ham-RGD)_2_ in mouse plasma

The in vitro stability of ^99m^Tc-(Ham-RGD)_2_ in mouse plasma was evaluated by incubating the probe in murine plasma at 37 °C for 60 and 180 min. The radiochemical purity of ^99m^Tc-(Ham-RGD)_2_ was 37.3 ± 3.7% (60 min) and 15.7 ± 5.8% (180 min), respectively.

### In vivo biodistribution

The uptake of ^99m^Tc-(Ham-RGD)_2_ in each organ was expressed as the %ID/g, except for that in the stomach and thyroid, which was expressed as the %ID (Table 2). ^99m^Tc-(Ham-RGD)_2_ was excreted mainly from the kidneys. The radioactivity of U87MG tumors (high Integrin ɑ_v_β_3_ expression) was significantly higher than that of PC3 tumors from 5 min after the administration of ^99m^Tc-(Ham-RGD)_2_. In addition, the accumulation in U87MG tumors was reduced by blocking with excess c(RGDfK) at 180 min after administration [6.75 ± 1.73%ID/g (non-blocking) vs. 4.00 ± 0.68%ID/g (blocking) (p < 0.01)], but there was no significant difference in accumulation in PC3 tumors between the non-blocking and blocking groups [1.30 ± 0.35%ID/g (non-blocking) vs. 1.29 ± 0.12%ID/g (blocking) (p = 1.00)] (Supplementary file, Table S1).

**Table 2:** Biodistribution of ^99m^Tc-(Ham-RGD)_2_ in the U87MG/PC3 tumor-bearing mice.

Organ	Time after injection			
	5 min	60 min	180 min	360 min
Blood	14.38 ± 0.83	9.77 ± 1.73	7.17 ± 1.46	5.90 ± 0.51
Spleen	2.72 ± 0.05	3.23 ± 0.57	3.01 ± 0.53	3.92 ± 0.63
Pancreas	1.60 ± 0.11	1.56 ± 0.30	1.34 ± 0.21	1.33 ± 0.25
Stomach^a^	1.07 ± 0.25	1.58 ± 0.28	1.62 ± 0.36	1.45 ± 0.19
Intestine	1.62 ± 0.11	3.51 ± 0.46	6.87 ± 1.06	10.62 ± 1.72
Kidney	6.93 ± 0.50	12.22 ± 1.78	10.98 ± 1.73	12.89 ± 1.88
Liver	4.05 ± 0.19	4.67 ± 1.04	4.12 ± 0.62	4.59 ± 0.72
Heart	3.33 ± 0.37	3.21 ± 0.48	2.44 ± 0.49	2.63 ± 0.39
Lung	7.84 ± 1.34	6.17 ± 1.12	5.14 ± 0.97	4.93 ± 0.72
Brain	0.35 ± 0.02	0.35 ± 0.10	0.28 ± 0.04	0.05 ± 0.01
Muscle	0.82 ± 0.17	1.04 ± 0.28	0.94 ± 0.26	0.91 ± 0.10
Thyroid^a^	0.04 ± 0.01	0.09 ± 0.02	0.06 ± 0.04	0.05 ± 0.01
U87MG	2.26 ± 0.71	6.80 ± 0.78	6.75 ± 1.73	10.01 ± 1.89
PC3	0.56 ± 0.09	1.08 ± 0.20	1.30 ± 0.35	1.36 ± 0.14

Values are expressed as the % injected dose per gram (%ID/g) of organ tissue. Each value is the mean ± standard deviation of 4–5 mice at each interval.

^a^Values are expressed as the % injected dose (%ID).

### SPECT imaging

**Figure 6:**
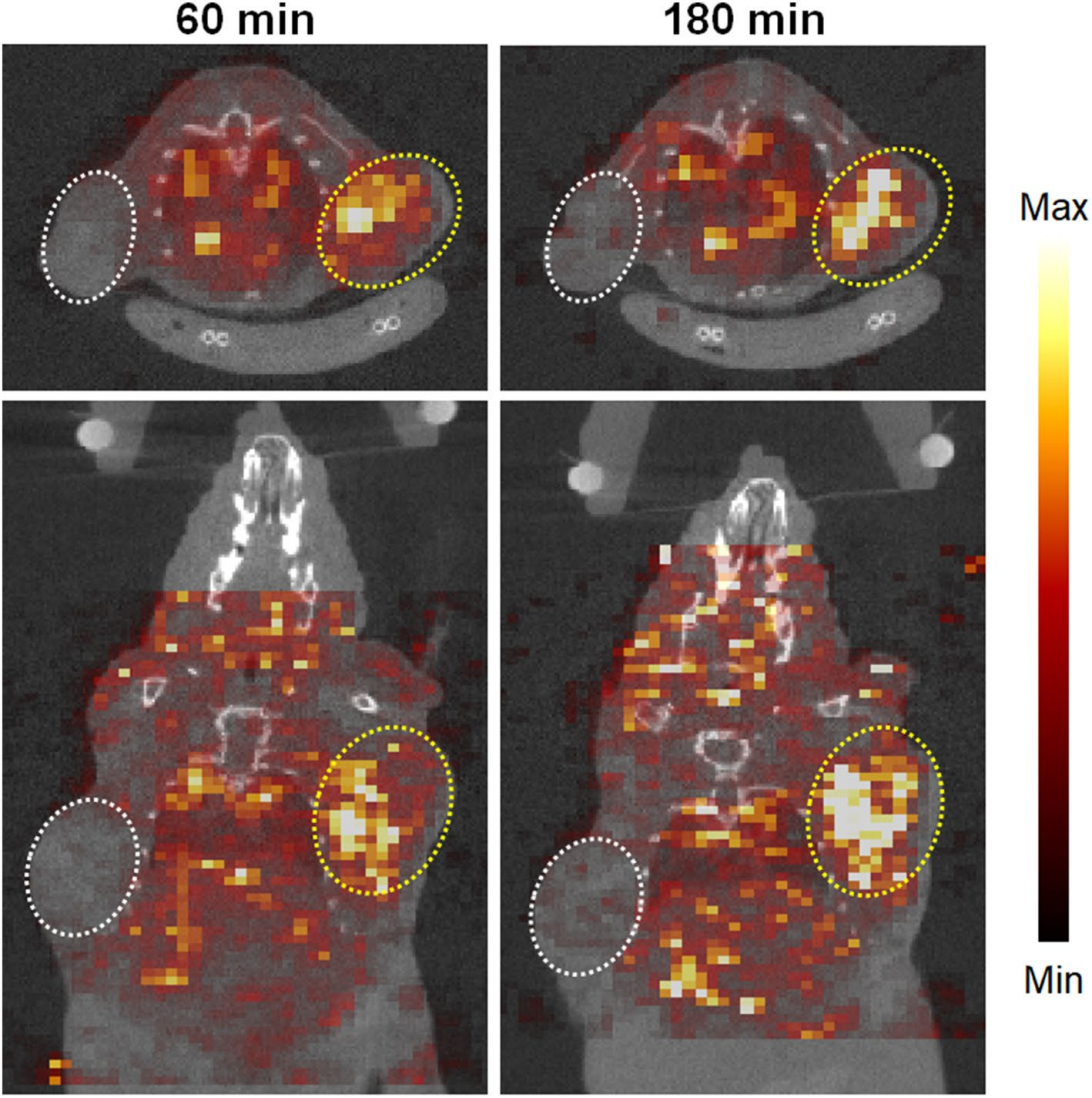
SPECT/CT images (upper: axial, lower: coronal) of the U87MG/PC3 tumor-bearing mouse after the administration of ^99m^Tc-(Ham-RGD)_2_. ^99m^Tc-(Ham-RGD)_2_ (2.8 MBq/0.1 mL saline) was injected intravenously into the mouse, and the SPECT imaging was acquired at 60 (left) and 180 min (right) after the administration. The CT images were acquired just before the SPECT imaging. The Yellow circle: U87MG tumor, white circle: PC3 tumor.

The SPECT/CT images were acquired at 60 and 180 min after the administration of ^99m^Tc-(Ham-RGD)_2_ (Fig. 6). The U87MG tumor was clearly visualized from 60 min, whereas the radioactive signal in the PC3 tumor was limited.

## Discussion

In this study, we designed and synthesized “Ham-Mal” as a novel Ham-based bifunctional chelating agent for ^99m^Tc. Ham-Mal was obtained through a four-step reaction from 4-aminobenzonitrile with a total yield of 7.2% (Fig. 2). We first converted the cyanide group of **1** to the Ham group directly; however, this reaction failed, possibly due to the instability of the maleimide group of **1**. Maleimide groups can be protected and deprotected with furan by the thermally reversible Diels–Alder click reaction^17^. Thus, the Ham group was introduced after protecting the maleimide group of **1**. After the synthesis of Ham-Mal, we conjugated ligands [cysteine and c(RGDfC)] having a thiol group with Ham-Mal via the thiol-maleimide reaction, and obtained Ham-Cys and Ham-RGD, respectively (Fig. 2).

Next, we performed the radiolabeling of Ham-Cys and Ham-RGD with ^99m^TcO_4_^-^. In general, the Ham group structure is known to have two tautomer, and thus the ^99m^Tc-Ham complex forms two tautomer which can be observed by RP-HPLC ^7^. The chelation of Ham and ^99m^Tc was confirmed by the disappearance of the peak from ^99m^TcO_4_^-^ (retention time: 2–4 min^11^) and then the appearance of two peaks because it is difficult to synthesize the analogues of the rhenium-Ham complex^7^. To search for an appropriate ^99m^Tc radiolabeling reaction condition, we first evaluated the reaction of Ham-Cys and ^99m^TcO_4_^-^ with different concentrations of Ham-Cys (final concentration: 10 and 20 mM) in different solvents [phosphate buffer (pH 7.4) and acetic acid/ethanol = 1/4]. In our previous study, we used an acetic acid/ethanol mixture as the solvent for ^99m^Tc radiolabeling reactions of compounds containing Ham^9–13^ to prevent forming by-products (especially, ^99m^Tc-colloid); however, the radiochemical yields were low [10.4 ± 9.8% (final concentration of Ham-Cys:10 mM), 11.0 ± 5.0% (20 mM)] (Table1) and the acquired ^99m^Tc-radiolabeled compound was unstable. On the other hand, ^99m^Tc radiolabeling reaction to give ^99m^Tc-(Ham-Cys)_2_ in phosphate buffer (pH 7.4) proceeded with radiochemical yields of 68.7 ± 2.4% (final concentration of Ham-Cys: 10 mM) and 75.3 ± 4.5% (20 mM) (Table 1). The acquired ^99m^Tc-radiolabeled compound was analyzed by RP-HPLC and only two radioactive peaks (retention times: 19.2 min and 20.6 min) were observed (Fig. 4A). Therefore, this suggested that the ^99m^Tc-radiolabeling reaction of compounds based on Ham-Mal proceeded in the neutral pH solvent. As for the concentration of the precursor, a final concentration of 10 mM was considered sufficient for the ^99m^Tc-radiolabeling reaction (Table 1). Thus, we performed the radiolabeling of Ham-RGD with ^99m^Tc using 10 mM (final concentration) Ham-RGD in phosphate buffer (pH 7.4), and acquired ^99m^Tc-(Ham-RGD)_2_ in a radiochemical yield of a 47% with a radiochemical purity of over 95% (Fig. 4B).

To confirm that ^99m^Tc-radiolabeled agents composed of Ham-Mal are able to target the biomolecules to which the ligands conjugated to Ham-Mal have a high affinity, we next performed in vitro and in vivo studies with ^99m^Tc-(Ham-RGD)_2_, and evaluated it as an Integrin ɑ_v_β_3_-targeting probe. Integrin ɑ_v_β_3_ is a well-known biomolecule that is closely related to the neovasculature and metastasis of cancer^18^. In addition, c(RGDfC), a targeting ligand of ^99m^Tc-(Ham-RGD)_2_, is known to have a high affinity to Integrin ɑ_v_β_3_^19^. In the in vitro study, the cellular uptake level of ^99m^Tc-(Ham-RGD)_2_ in U87MG cells (high Integrin α_v_β_3_ expression) was significantly higher than that in PC3 cells (low Integrin ɑ_v_β_3_ expression) (U87MG: 1.87 ± 0.28 vs. PC3: 0.49 ± 0.08%dose/mg protein, p < 0.01) (Fig. 5). In addition, the uptake level of ^99m^Tc-(Ham-RGD)_2_ in U87MG cells was significantly reduced by pretreatment with excess non-radiolabeled cyclic RGD peptide [c(RGDfK)] (0.95 ± 0.04%dose/mg protein, p < 0.05) (Fig. 5). This suggests that ^99m^Tc-(Ham-RGD)_2_ was taken up specifically by cells with high Integrin ɑ_v_β_3_ expression.

In the in vivo biodistribution study, ^99m^Tc-(Ham-RGD)_2_ also accumulated highly in U87MG tumors compared with PC3 tumors from 5 min after its i.v. administration (Table 2). In addition, the accumulation level in U87MG tumors was reduced by blocking with excess non-radiolabeled c(RGDfK) at 180 min after administration [4.00 ± 0.68%ID/g (blocking) (p < 0.05)] (Supplementary file, Table S1), which suggests that ^99m^Tc-(Ham-RGD)_2_ accumulated specifically in tumors with high Integrin ɑ_v_β_3_ expression. We also performed SPECT/CT imaging study of ^99m^Tc-(Ham-RGD)_2_, and confirmed that ^99m^Tc-(Ham-RGD)_2_ can selectively visualize U87MG tumors, consistent with the in vivo biodistribution study (Fig. 6). These results support the use of ^99m^Tc-(Ham-RGD)_2_ as a SPECT imaging probe targeting tumors with high Integrin ɑ_v_β_3_ expression. In the in vivo biodistribution study, we also observed high radioactivity in the kidneys (Table 2). This may be due to the properties of c(RGDfC) because Integrin ɑ_v_β_3_ was reported to be expressed in the kidneys, especially in glomerular epithelial cells and Bowman's capsule^20^, and that the conventional agents based on RGD peptides accumulated highly in the kidneys^21^. As for the stomach and thyroid where free ^99m^Tc (^99m^TcO_4_^−^) is known to accumulate highly^22^, less radioactivity was observed at 360 min after the administration of ^99m^Tc-(Ham-RGD)_2_ (Table 2). We also evaluated the in vitro stability of ^99m^Tc-(Ham-RGD)_2_ in mouse plasma; however, we found that the radiochemical purity decreased in a time dependent manner, and some unknown hydrophilic metabolites emerged (Figure S2). Given that other Ham-based ^99m^Tc radiolabeled compounds we developed previously^11,13^ showed high stability in plasma and good blood clearance, it is suspected that those unknown metabolites affected the slow blood clearance. Although the metabolites and the reason of the instability is still unclear, the low stability of ^99m^Tc-(Ham-RGD)_2_ in plasma is the limitation, and further study for redesigning the bifunctional chelate to improve the stability would be needed for use it in in vivo study.

To date, several chelating groups, such as D-penicillamine^23,24^ and isocyanide^25,26^, have been reported to form multivalent complexes with ^99m^Tc. These chelates reacted with ^99m^Tc effectively at a low concentration of the precursors, but their reaction temperature was high (100–120 °C). On the other hand, the precursors based on Ham-Mal reacted with ^99m^Tc under moderate conditions (at room temperature), which is suitable for radiolabeling thermally instable compounds such as proteins (e.g. antibody and its fragment). For radiolabeling ^99m^Tc with those thermally instable compounds, some chelates such as hydrazinonicotinamide (HYNIC) have been used^27,28^. Given that these chelates form ^99m^Tc-complexes with a metal-to-ligand ratio of 1:1, Ham has an advantage in terms of developing ^99m^Tc-labeled compounds with a bivalent targeting scaffold, so that Ham-Mal would be a promising bifunctional chelate for developing those ^99m^Tc-labeled compounds.

In this study, we designed and synthesized a novel bifunctional ^99m^Tc-chelating agent based on Ham, “Ham-Mal”. ^99m^Tc radiolabeled agents were acquired easily by reacting Ham-Mal and the targeting ligands via thiol-maleimide reaction, and then by the ^99m^Tc-radiolabeling reaction under moderate conditions. The acquired ^99m^Tc radiolabeled agent [^99m^Tc-(Ham-RGD)_2_ in this study]demonstrated good visualization of specific organs expressing the target biomolecule. This study suggests that Ham-Mal is available for the development of ^99m^Tc-radiolabeled SPECT imaging probes containing bivalent targeting ligands.

## Methods

### Materials

All reagents were obtained commercially and used without further purification unless otherwise indicated. ^99m^Tc-NaTcO_4_ was purchased from Nihon Medi-Physics Co., Ltd. (Tokyo, Japan). W-Prep 2XY (Yamazen, Osaka, Japan) was used for silica gel column chromatography on a Hi Flash silica gel column (40 μm, 60 Å, Yamazen). ^1^H NMR and ^13^C NMR spectra were recorded on a JNM-ECS400 (JEOL, Tokyo, Japan) with tetramethylsilane as an internal standard. Coupling constants are reported in Hertz. Multiplicity was defined as singlet (s), doublet (d), or multiplet (m). High-resolution mass spectrometry (HRMS) of fast atom bombardment (FAB) and electrospray ionization (ESI) were carried out with a JEOL JMS-700 (JEOL) and a LCMS-IT-TOF (SHIMADZU, Kyoto, Japan), respectively. High-performance liquid chromatography (HPLC) was performed with a Shimadzu system (SHIMADZU, an LC-20AT pump with an SPD-20A UV detector, λ = 254 nm).

### Chemistry

#### Synthesis of 1

A solution of 4-aminobenzonitrile (2.36 g, 20 mmol) and maleic anhydride (5.88 g, 60 mmol) in 1,4-dioxane (30 mL) was stirred at 100 °C for 1 h. To the reaction mixture, ammonium persulfate (9.12 g, 40 mmol) and dimethyl sulfoxide (DMSO) (2.84 mL) were added and the reaction mixture was heated to 100 °C for 3 h. Thereafter, the reaction mixture was filtered and dioxane was removed under vacuo. The residue was dissolved in ethyl acetate and washed with diluted HCl, saturated aqueous NaHCO_3_, and brine. The organic layer was dried over Na_2_SO_4_ and the solvent was evaporated under vacuo to afford 3.38 g of **1** (yield: 85%). ^1^H NMR (400 MHz, DMSO-d_6_) *δ* 7.25 (s, 2H), 7.59 (d, *J* = 8.4 Hz, 2H), 7.98 (d, *J* = 8.8 Hz, 2H). ^13^C NMR (100 MHz, DMSO-d_6_) δ 109.88, 118.49, 126.86, 133.06, 135.02, 135.88, 169.34.

#### Synthesis of 2

A solution of **1** (3.31 g, 16.7 mmol) in acetonitrile (10 mL) was mixed with furan (5 mL) and stirred at 60 °C for 8 h. The solvent was evaporated and ethyl acetate was added, then a solid precipitated out. The material was filtered and washed with ethyl acetate. The filtrated solution was concentrated to afford more of the product, which was also filtered and washed with ethyl acetate. The combined solid portions were dried under high vacuum at room temperature overnight to afford 2.67 g of **2** (yield: 60%). ^1^H NMR (400 MHz, DMSO-*d*_*6*_) *δ* 3.13 (s, 2H), 5.26 (s, 2H), 6.62 (s, 2H), 7.47 (d, *J* = 8.0 Hz, 2H), 7.98 (d, *J* = 8.0 Hz, 2H). ^13^C NMR (100 MHz, DMSO-d_6_) δ 47.71, 80.91, 110.98, 118.29, 127.55, 133.19, 136.05, 136.72, 175.27. HR-MS (FAB, pos) *m/z* calcd. for C_15_H_11_N_2_O_3_^+^, 267.0770; found 267.0768 [M + H]^+^.

#### Synthesis of 3

A solution of hydroxylamine hydrochloride (521 mg, 7.5 mmol) and triethylamine (1.04 mL, 7.5 mmol) in *N*,*N*-dimethylformamide (DMF) (5 mL) and H_2_O (2 mL) was added to a solution of **2** (1.33 g, 5 mmol) in DMF (10 mL). The reaction mixture was stirred at room temperature for 3 h. Then, H_2_O was added to the solution and diluted with ethyl acetate. The organic layer was dried over Na_2_SO_4_ and the solvent was evaporated under vacuo. The residue was recrystallized with chloroform and dried under high vacuum at room temperature overnight to afford 287 mg of **3** (yield: 19%). ^1^H NMR (400 MHz, DMSO-*d*_*6*_) δ 3.08 (s, 2H), 5.24 (s, 2H), 5.88 (s, 2H), 6.60 (s, 2H), 7.19 (d, *J* = 8.4 Hz, 2H), 7.74 (d, *J* = 8.4 Hz, 2H), 9.74 (s, 1H). ^13^C NMR (100 MHz, DMSO-*d*_*6*_) δ 47.51, 80.80, 126.01, 126.46, 132.41, 133.41, 136.66, 150.27, 175.68. HR-MS (FAB, pos) *m/z* calculated for C_15_H_14_N_3_O_4_^+^, 300.0984; found 300.0985 [M + H]^+^.

### Synthesis of 4 (Ham-Mal)

A solution of **3** (299 mg, 1 mmol) in DMF (6 mL) and toluene (18 mL) was stirred at 115 ºC for 2 h. After evaporation of the solvent, the residue was suspended in H_2_O and then filtered. The solid portion was dried under high vacuum at room temperature overnight to afford 231 mg of **4** (yield: 74%). ^1^H NMR (400 MHz, DMSO-*d*_*6*_) δ 5.87 (s, 2H), 7.20 (s, 2H), 7.33 (d, *J* = 8.4 Hz, 2H), 7.75 (d, *J* = 8.8 Hz, 2H), 9.72 (s, 1H). ^13^C NMR (100 MHz, DMSO-*d*_*6*_) δ 125.85, 126.28, 131.94, 132.65, 134.73, 150.29, 169.82. HR-MS (ESI, pos) *m/z* calculated for C_11_H_10_N_3_O_3_^+^, 232.0722; found 232.0725 [M + H]^+^.

### Synthesis of 5 (Ham-Cys)

A solution of L-cysteine (10 mg, 80 µmol) in H_2_O (100 µL) was adjusted to pH 7–8 by 0.1 M NaOH and then added to a solution of **4** (10 mg, 40 µmol) in DMF (500 µL). The reaction mixture was stirred at room temperature for 1 h. Then, the solvent was evaporated and the residue was purified by RP-HPLC on a Cosmosil 5C_18_-AR-II column (Nacalai Tesque, Kyoto, Japan, diameter: 10 mm, length: 250 mm, particle size: 5 μm, pore size: 120 Å) using the mobile phase [H_2_O (0.1% TFA)/acetonitrile (0.1% TFA) = 90/10] at a flow rate of 4.0 mL/min to give 3.5 mg of **5** (yield: 23%). ^1^H NMR (400 MHz, D_2_O) δ 3.07–3.14 (m, 2H), 3.24–3.34 (m, 2H), 4.08–4.18 (m, 2H), 7.39 (d, *J* = 8.0 Hz, 2H), 7.70 (d, *J* = 8.0 Hz, 2H). ESI-HRMS *m/z* calculated for C_14_H_17_N_4_O_5_S^+^, 353.0920; found, 353.0919 [M + H]^+^.

### Synthesis of 6 (Ham-RGD)

A solution of c(RGDfC) (PEPTIDE INSTITUTE, INC., Osaka, Japan, 2.0 mg, 4 µmol) in H_2_O (200 µL) was added to a solution of **4** (0.8 mg, 4 µmol) in DMF (500 µL). The reaction mixture was stirred at room temperature for 1 h. Then, the solvent was evaporated and the residue was purified by RP-HPLC on a Cosmosil 5C_18_-AR-II column (Nacalai Tesque, diameter: 10 mm, length: 250 mm, particle size: 5 μm, pore size: 120 Å) using the mobile phase [H_2_O (0.1% TFA)/acetonitrile (0.1% TFA) = 90/10 (0 min) to 70/30 (30 min)] at a flow rate of 4.0 mL/min to give 1.8 mg of **6** (64% yield). HR-MS (ESI, pos) *m/z* calculated 810.2993, found 810.2988 [M + H]^+^.

### Radiolabeling of ^99m^Tc-(Ham-Cys)_2_

To a solution of **5** (Ham-Cys, final concentration: 10 mM or 20 mM) in an acetic acid/ethanol mixture (1/4, 50 μL) or phosphate buffer (pH 7.4, 10 mM, 50 µL), 100 μL of ^99m^Tc-NaTcO_4_ solution (18–30 MBq) and 15 μL of 3 mM tin(II) tartrate hydrate solution in H_2_O were added. The reaction mixture was incubated at room temperature for 10 min and purified by RP-HPLC on a YMC-Triart C18 (YMC CO., LTD., Kyoto, Japan, diameter: 4.6 mm, length: 150 mm, particle size: 5 μm, pore size: 120 Å) using the mobile phase [10 mM phosphate buffer (pH 7.4)/acetonitrile = 100/0 (0 min) to 70/30 (30 min)] at a flow rate of 1.0 mL/min. The two peaks (retention time: around 19 to 20 min, and around 20 to 21 min) were collected, mixed, and used as ^99m^Tc-(Ham-Cys)_2_ in the following study. The radiochemical yield of ^99m^Tc-(Ham-Cys)_2_ was calculated by dividing the radioactivity of the collected two peaks by the radioactivity of the reaction mixture measured just before the HPLC purification, in accordance with our previous study^11^. The acquired ^99m^Tc-(Ham-Cys)_2_ was analyzed by RP-HPLC using the same method described above. The radiochemical purity was calculated by dividing the radioactivity of the two peaks derived from the ^99m^Tc-(Ham-Cys)_2_ by the total radioactivity acquired from the HPLC chart (Fig. 4A).

### Radiolabeling of ^99m^Tc-(Ham-RGD)_2_

To a solution of **6** (Ham-RGD, final concentration: 10 mM) in phosphate buffer (pH 7.4, 10 mM, 50 µL), 100 μL of ^99m^Tc-NaTcO_4_ solution (18–33 MBq) and 15 μl of 3 mM tin(II) tartrate hydrate solution in H_2_O were added. The reaction mixture was incubated at room temperature for 10 min and purified by RP-HPLC on a YMC-Triart C18 (YMC CO., LTD., diameter: 4.6 mm, length: 150 mm, particle size: 5 μm, pore size: 120 Å) using the mobile phase [10 mM phosphate buffer (pH 7.4)/acetonitrile = 80/20 (0 min) to 60/40 (30 min)] at a flow rate of 1.0 mL/min. The two peaks (retention time: around 13 min, and around 15 min) were collected, mixed, and used as ^99m^Tc-(Ham- RGD)_2_ in the following study. The radiochemical yield of ^99m^Tc-(Ham-RGD)_2_ was calculated by dividing the radioactivity of the collected two peaks by the radioactivity of the reaction mixture measured just before the HPLC purification, in accordance with our previous study^11^. The acquired ^99m^Tc-(Ham-RGD)_2_ was analyzed by RP-HPLC using the same method described above. The radiochemical purity was calculated by dividing the radioactivity of the two peaks derived from the ^99m^Tc-(Ham- RGD)_2_ by the total radioactivity acquired from the HPLC chart (Fig. 4B).

### Cellular uptake study of ^99m^Tc-(Ham-RGD)_2_

According to the previously reported study, U87MG human glioblastoma cells (DS Pharma Biomedical, Osaka, Japan) and PC3 human prostate carcinoma cells (DS Pharma Biomedical) were used as Integrin ɑ_v_β_3_ high- and low-expressing cells, respectively^29^. U87MG and PC3 cells were maintained in Dulbecco’s modified Eagle’s medium (DMEM) or Roswell Park Memorial Institute 1640 (RPMI 1640) containing 10% fetal bovine serum, and 100 U/mL of penicillin and streptomycin in a 5% CO_2_ incubator at 37 ºC. These cells were sub-cultured overnight in 12-well plates (4 × 10^5^ cells/1 mL), and then pre-incubated in DMEM (1 mL) with or without c(RGDfK) (final concentration: 10 µM) for 30 min at 37 ºC in a humidified atmosphere containing 5% CO_2_. After the pre-incubation, the medium was exchanged with DMEM containing ^99m^Tc-(Ham-RGD)_2_ (74 kBq, 1 mL) and then incubated for 60 min. After the incubation, the cells were washed twice with phosphate-buffered saline (PBS) (1 mL) and then lysed with 1 M NaOH (0.4 mL). The radioactivity in the lysates was measured by a Wallac WIZARD 2470 gamma counter (PerkinElmer, Waltham, MA, USA) and total protein concentrations in samples were measured using the bicinchonic acid method.

### In vitro stability of ^99m^Tc-(Ham-RGD)_2_ in mouse plasma

The in vitro stability of ^99m^Tc-(Ham-RGD)_2_ in mouse plasma was performed in accordance to our previous study with some modification^11^. In brief, ^99m^Tc-(Ham-RGD)_2_ (about 500 kBq) was added to prepared fresh murine plasma (200 μL) collected from ddY mice (male, 5 weeks old). After incubating the solution at 37 °C for 60 and 180 min, acetonitrile (200 μL) was added. Subsequently, the solution was centrifuged (4000*g*, 10 min) and the supernatant was analyzed using RP-HPLC on a YMC-Triart C18 (YMC CO., LTD., diameter: 4.6 mm, length: 150 mm, particle size: 5 μm, pore size: 120 Å) using the mobile phase [10 mM phosphate buffer (pH 7.4)/acetonitrile = 80/20 (0 min) to 60/40 (30 min)] at a flow rate of 1.0 mL/min.

### In vivo biodistribution of ^99m^Tc-(Ham-RGD)_2_

Animal experiments were approved by the Kyoto University Animal Care Committee, and performed in accordance with ARRIVE guidelines (https://arriveguidelines.org) and the institutional guidelines for animal care of Kyoto University. BALB/c athymic nude mice (male, 5 weeks) supplied by Japan SLC, Inc. (Hamamatsu, Japan) were housed under a 12-h light/12-h dark cycle, and given free access to food and water. PC3 cells [5 × 10^6^ cells/0.1 mL of RPMI1640 and Geltrex (Thermo Fisher Scientific, Waltham, MA, USA) at a ratio of 1:1] were subcutaneously inoculated into the left flank of mice. At 2 weeks after the inoculation of PC3 cells, U87MG cells [5 × 10^6^ cells/0.1 mL of DMEM and Geltrex (Thermo Fisher Scientific) at a ratio of 1:1] were subcutaneously inoculated into the right flank of mice, and a biodistribution study was performed after a 2-week growth period. ^99m^Tc-(Ham-RGD)_2_ (370 kBq/0.1 mL saline) was injected intravenously into the tumor-bearing mice. Mice were sacrificed by decapitation at 5, 60, 180, and 360 min after administration. The tumor, blood, muscle, and other organs were then excised, and their radioactivity and weight were measured. For the blocking study, c(RGDfK) (10 mg/kg weight) dissolved in 0.1 mL of saline was injected intravenously into the tumor-bearing mice. At 15 min after the injection of c(RGDfK), ^99m^Tc-(Ham-RGD)_2_ (370 kBq/0.1 mL saline) was injected intravenously into the mice, which were sacrificed by decapitation at 180 min after administration. The tumor, blood, muscle, and other organs were then excised, and their radioactivity and weight were measured.

### SPECT imaging

SPECT/CT imaging study was performed using a Triumph LabPET12/SPECT4/CT (TriFoil Imaging Inc., Chatsworth, CA, USA) by the previously reported method with modification^30^. ^99m^Tc-(Ham-RGD)_2_ (2.8 MBq/0.1 mL saline) was injected intravenously into the tumor-bearing mice prepared by the above method. At 45 and 165 min after administration, the mice were anesthetized with 1.5–2.0% isoflurane for approximately 10 min, and then CT imaging was performed with X-ray sources set to 60 kVp and 360 μA. After CT imaging at 60 and 180 min after the administration of ^99m^Tc-(Ham-RGD)_2_, SPECT imaging was performed with multi-pinhole collimators (1.0 mm diameter) and the projection data were acquired using a 20% energy window centered at 140 keV for ^99m^Tc, with a 360° circular orbit, 60-s projection time, and 32 projection angles. The acquired SPECT data were reconstructed by a three-dimensional ordered subset expectation maximization (OSEM) algorithm with CT-based attenuation correction and then analyzed using Amide software (Amide.exe ver. 1.0.4, SourceForge.net, https://sourceforge.net/projects/amide/files/amide/1.0.4/).

### Statistics

Data are presented as the mean ± S.E.M (for the in vitro study) or the mean ± S.D (for the in vivo study). In the in vitro study, statistical analyses were performed using 2-way ANOVA following the Tukey–Kramer test with JMP 14 software (SAS Institute Inc., Cary, NC, USA). For the in vivo study, the Student’s *t*-test was used to assess the significance of differences. Differences at the 95% confidence level (P < 0.05) were considered significant.

## Supplementary Information

Supplementary Information.
