# Supplementary material for: Development of a hydroxamamide-based bifunctional chelating agent to prepare technetium-99m-labeled bivalent ligand probes
Source: Sci Rep. 2021 Sep 21;11:18714. doi: 10.1038/s41598-021-98235-x (PMC8455562; doi:10.1038/s41598-021-98235-x)

Supplementary file

Development of a hydroxamamide-based bifunctional chelating agent to prepare technetium-99m-labeled bivalent ligand probes

Yoichi Shimizu[1,2,#,*], Masato Ando[1,#], Shimpei Iikuni[1], Hiroyuki Watanabe[1], and Masahiro Ono[1*]

[1] Department of Patho-Functional Bioanalysis, Graduate School of Pharmaceutical Sciences, Kyoto University, 46-29, Yoshida Shimoadachi-cho, Sakyo-ku, Kyoto 606-8501, Japan

[2] Department of Diagnostic Imaging and Nuclear Medicine, Graduate School of Medicine, Kyoto University, 54 Kawahara-cho, Shogoin, Sakyo-ku, Kyoto 606-8507, Japan

* Corresponding author:
Yoichi Shimizu. Address: 54 Shogoinkawahara-cho, Sakyo-ku, Kyoto 606-8507, Japan, Phone: +81-75-751-3760, Fax: +81-75-771-9709, E-mail: yoichis@kuhp.kyoto-u.ac.jp
Masahiro Ono. Address: 46-29, Yoshida Shimoadachi-cho, Sakyo-ku, Kyoto 606-8501, Japan, Phone: +81-75-753-4556, Fax: +81-75-753-4568, E-mail: ono@pharm.kyoto-u.ac.jp

[#]Y.S. and M.A. contributed equally to this manuscript.

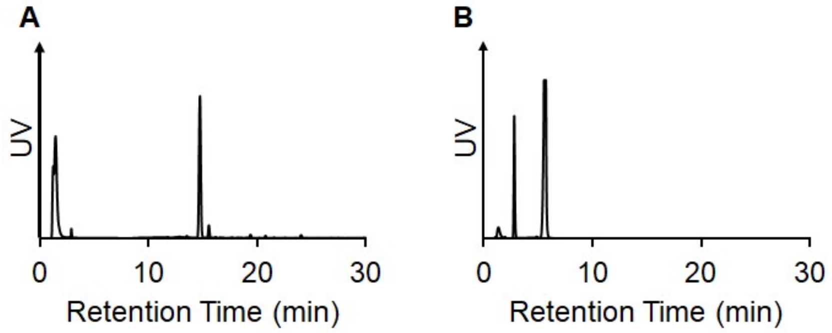

Figure S1. Chromatograms for the solution containing Ham-Cys (A) and Ham-RGD (B). The retention times of Ham-Cys and Ham-RGD were 14.7 and 5.7 min, respectively.

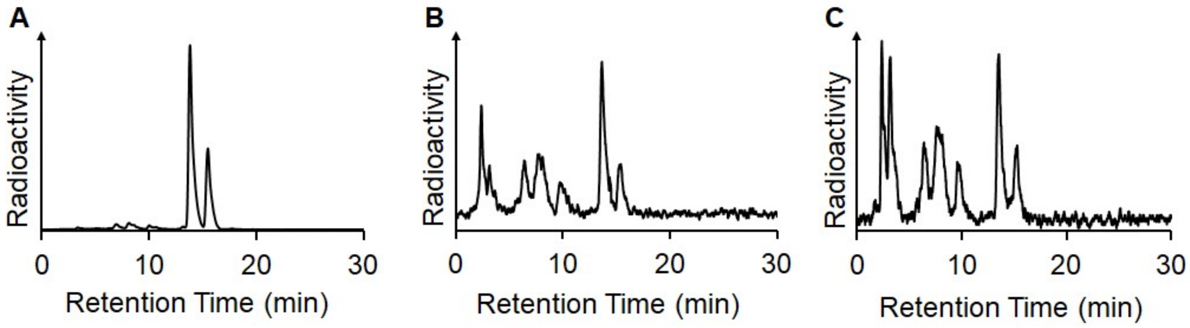

Figure S2. Radiochromatograms of ^{99m}Tc-(Ham-RGD)$_2$ before (A) or after incubation in mouse plasma for 60 min (B) or 180 min (C).

Table S1. The radioactivity of each organ of the U87MG/PC3 tumor-bearing mice with or without blocking by c(RGDfK) at 180 min after administration of ^{99m}Tc-(Ham-RGD)$_2$.

Organ	Non-blocking	Blocking
Blood	7.17 ± 1.46	6.97 ± 0.71
Spleen	3.01 ± 0.53	1.75 ± 0.17**
Pancreas	1.34 ± 0.21	0.89 ± 0.06**
Stomach#	1.62 ± 0.36	1.47 ± 0.40
Intestine	6.87 ± 1.06	4.54 ± 1.12*
Kidney	10.98 ± 1.73	6.71 ± 0.44**
Liver	4.12 ± 0.62	2.74 ± 0.20**
Heart	2.44 ± 0.49	2.13 ± 0.19
Lung	5.14 ± 0.97	4.35 ± 0.72
Brain	0.28 ± 0.04	0.33 ± 0.25
Muscle	0.94 ± 0.26	0.68 ± 0.09
Thyroid#	0.06 ± 0.04	0.05 ± 0.01
U87MG	6.75 ± 1.73	4.00 ± 0.68*
PC3	1.30 ± 0.35	1.29 ± 0.12

Values are expressed as the % injected dose per gram (%ID/g) of organ tissue. Each value is the mean ± standard deviation of 4-5 mice at each interval. #Values are expressed as the % injected dose (%ID). *$p < 0.05$, **$p < 0.01$ vs. non-blocking group.

Compound **1** (^{1}H-NMR)

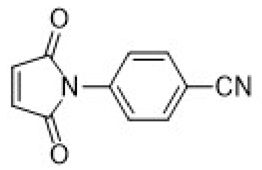

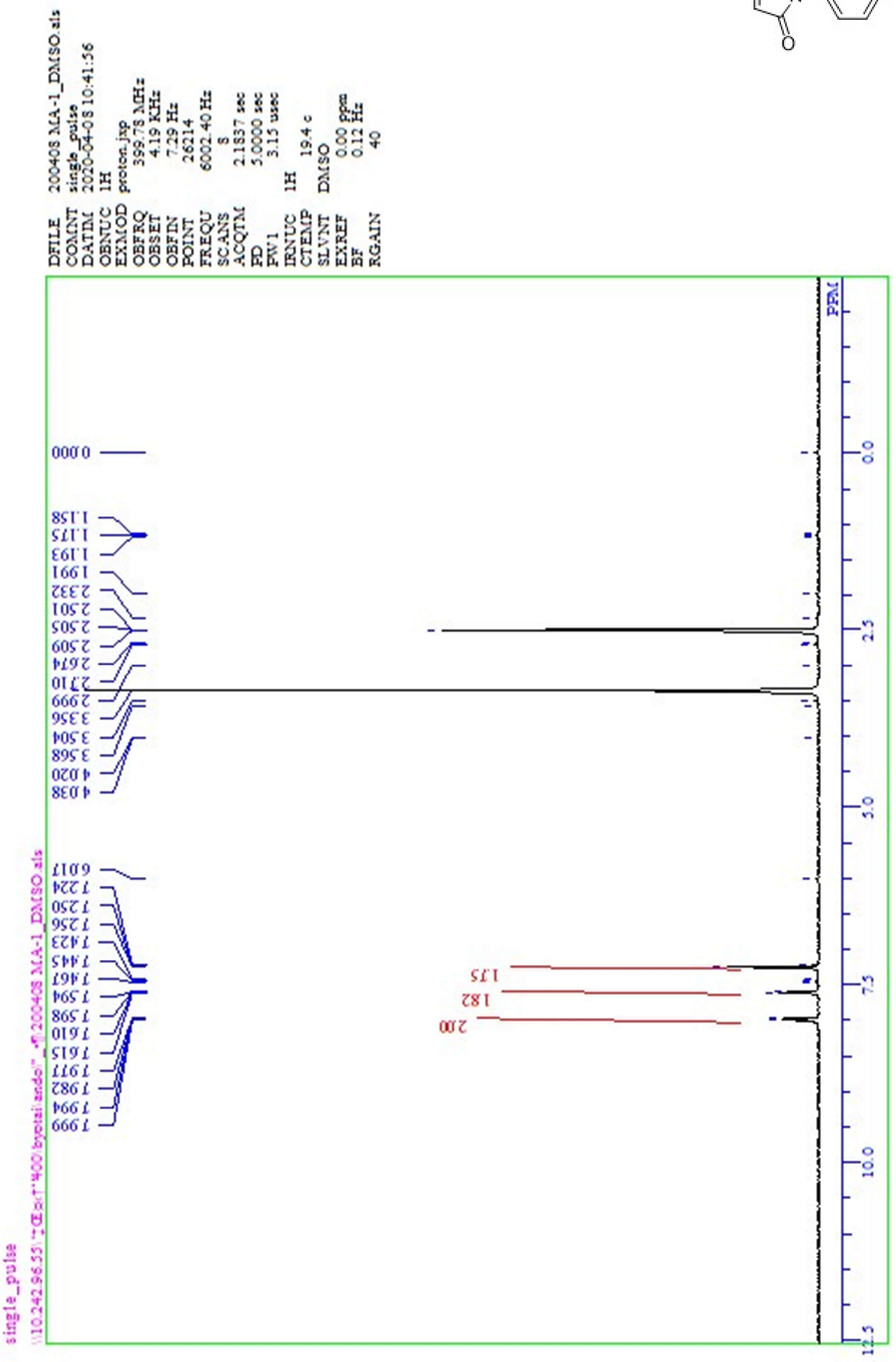

Compound **1** (^{13}C-NMR)

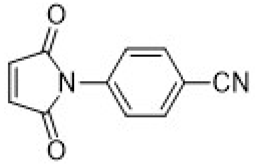

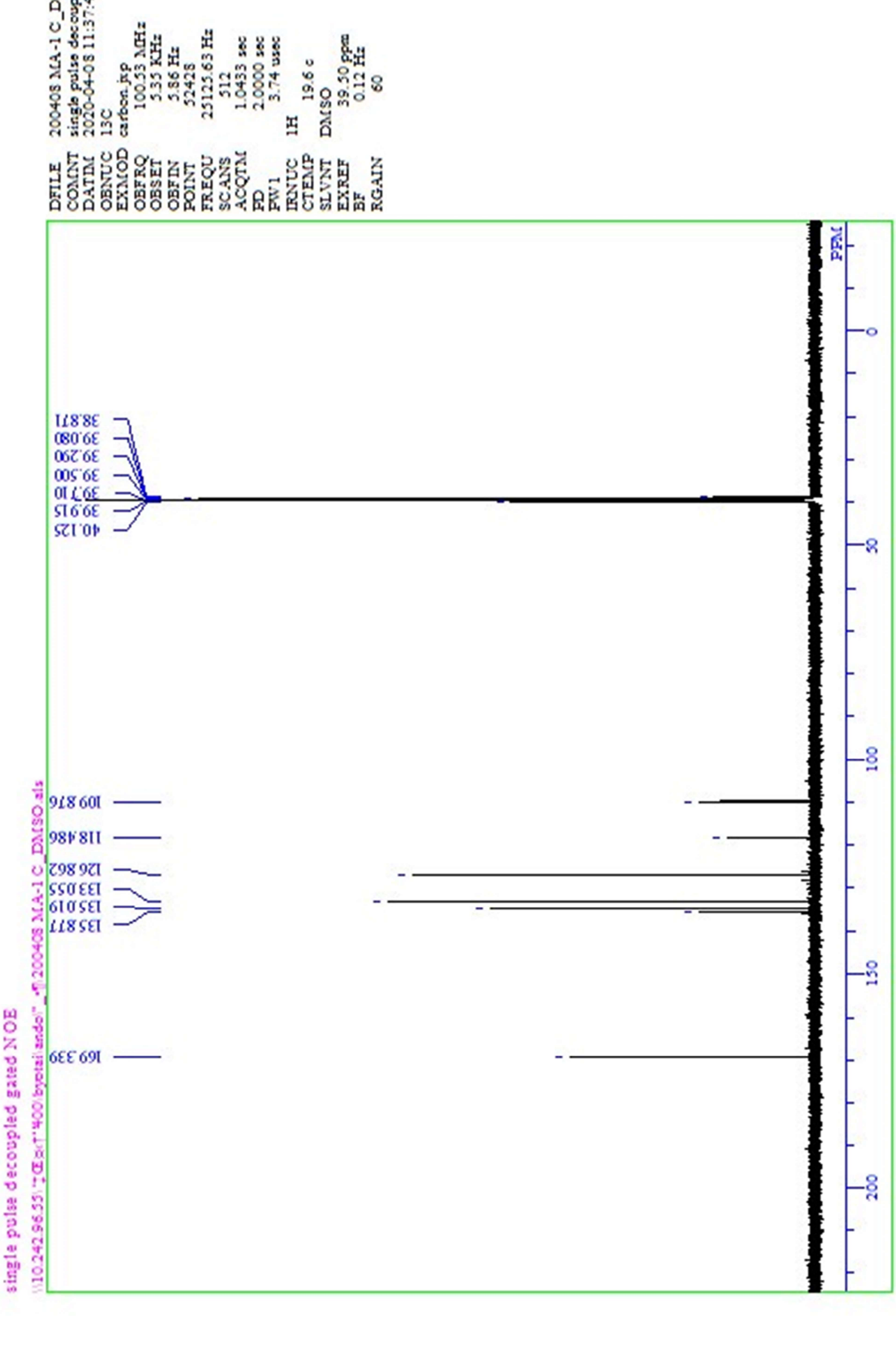

Compound **2** (^{1}H-NMR)

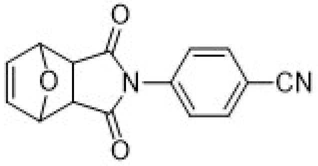

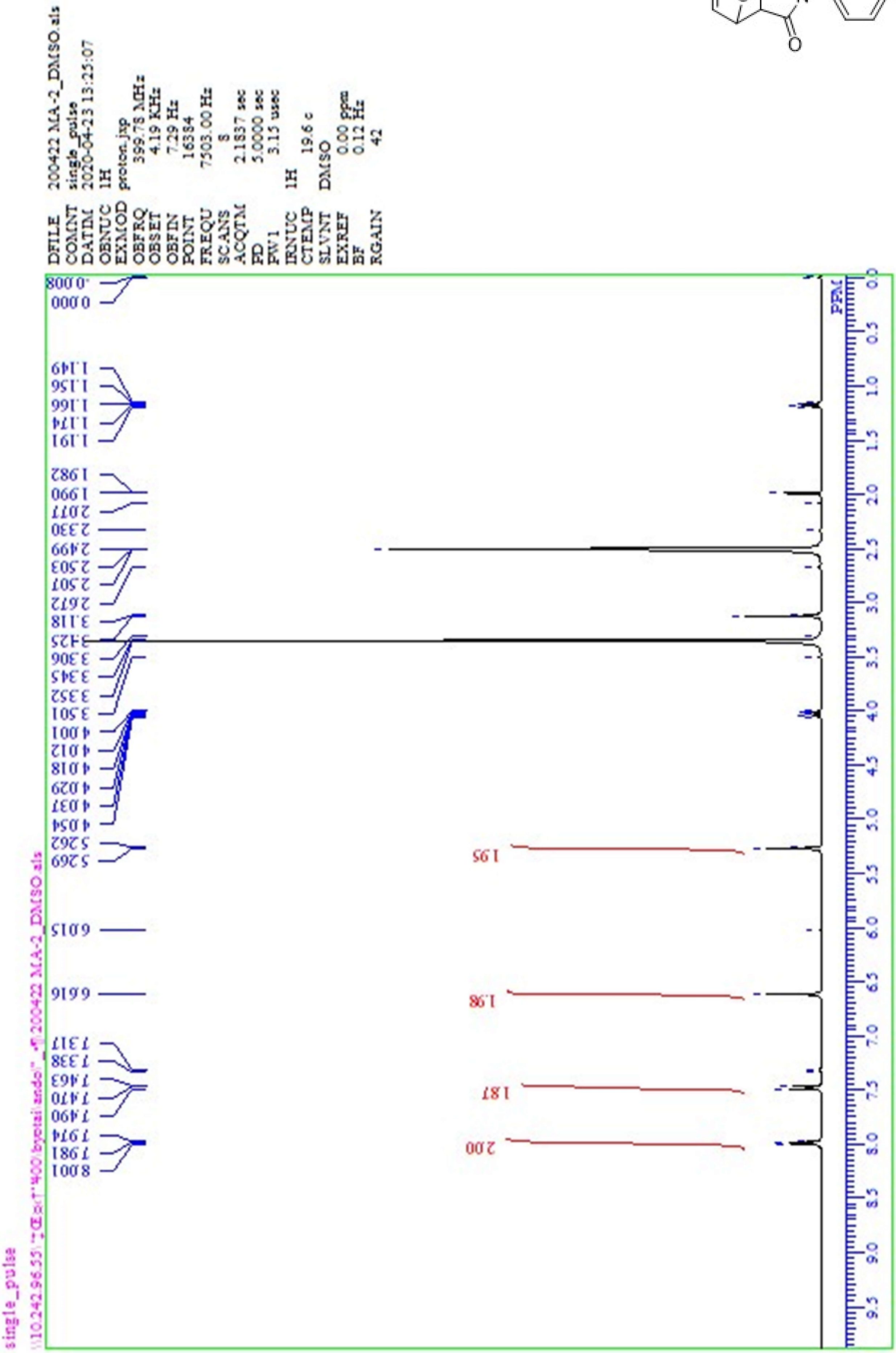

Compound **2** (^{13}C-NMR)

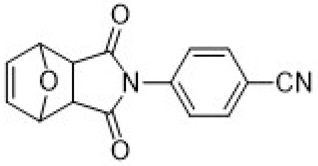

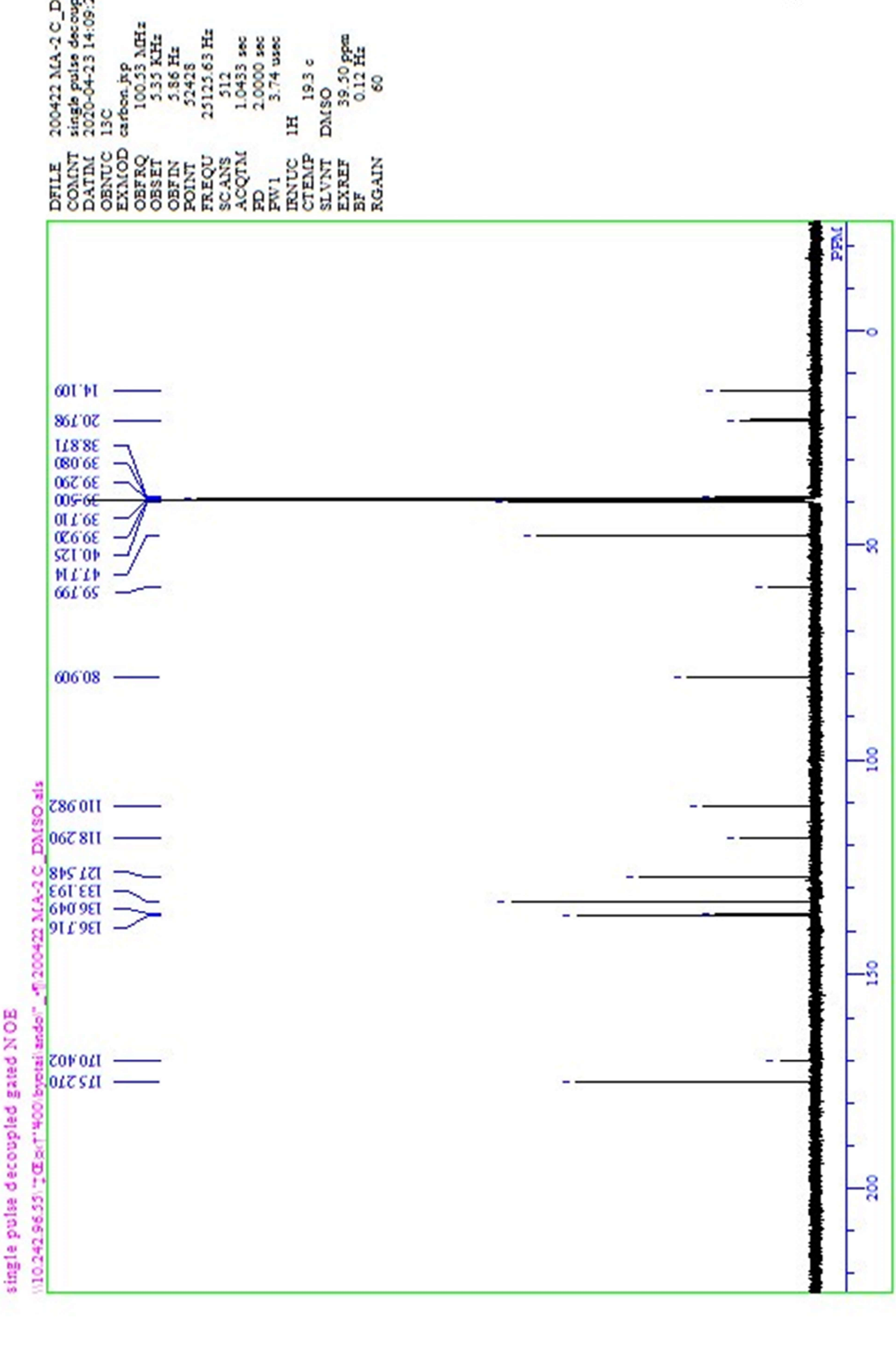

Compound **3** (^{1}H-NMR)

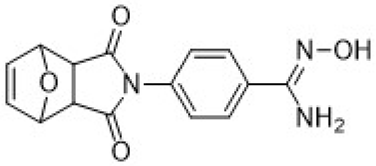

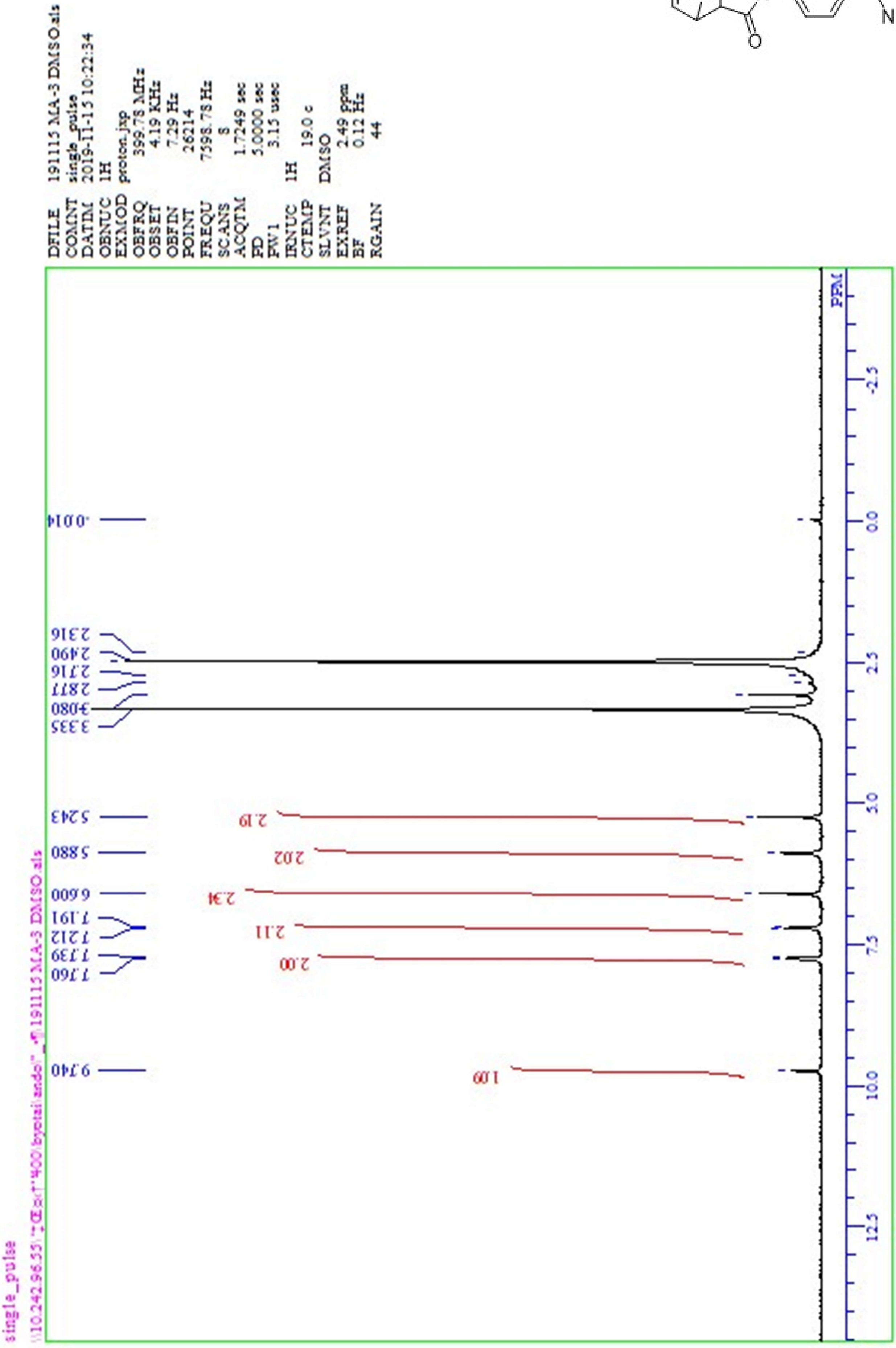

Compound **3** (^{13}C-NMR)

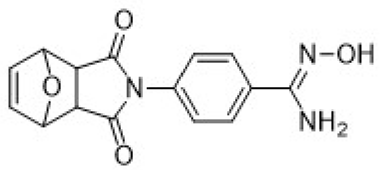

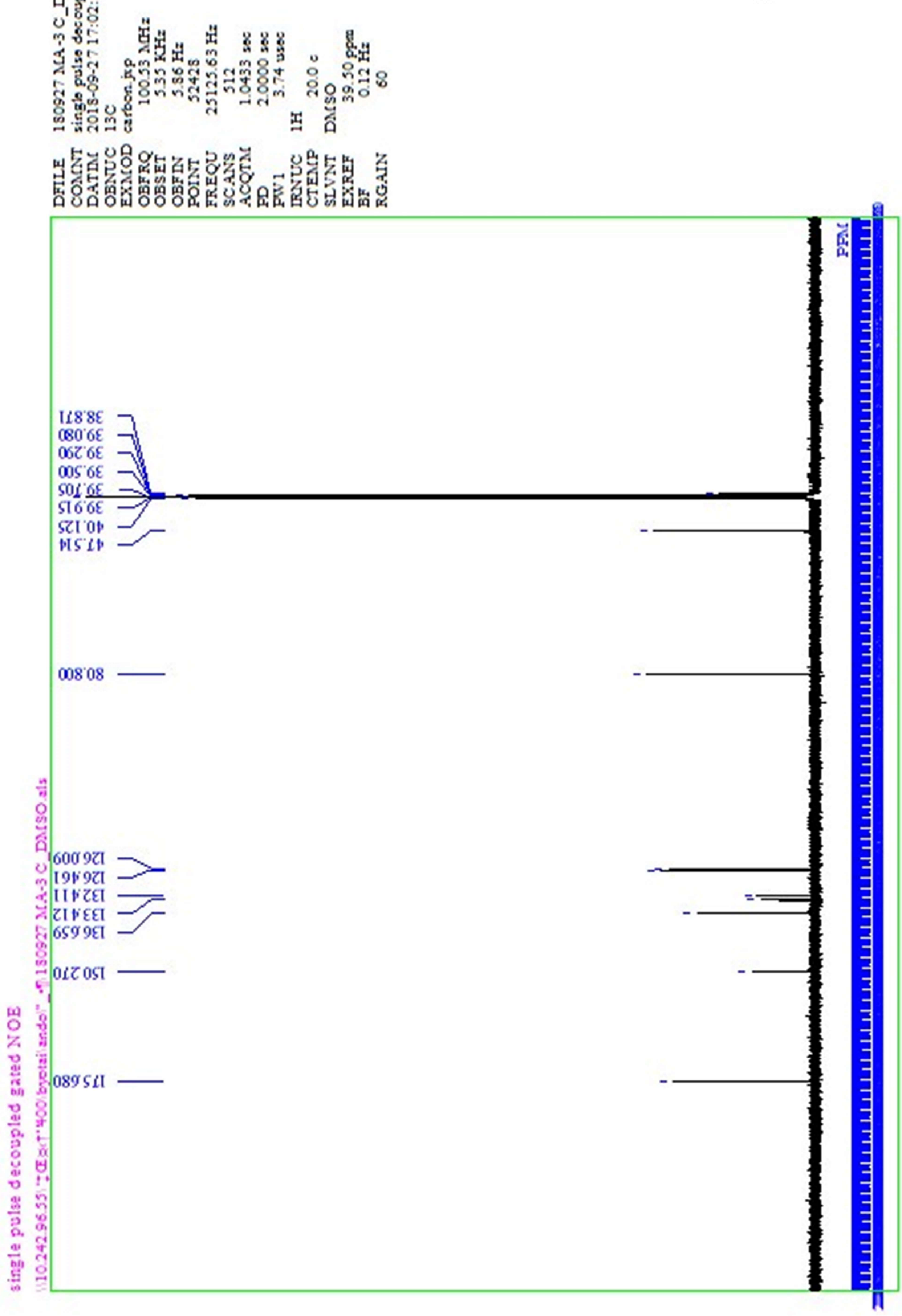

Ham-Mal (Compound **4)** (^{1}H-NMR)

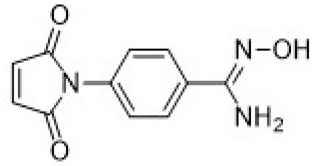

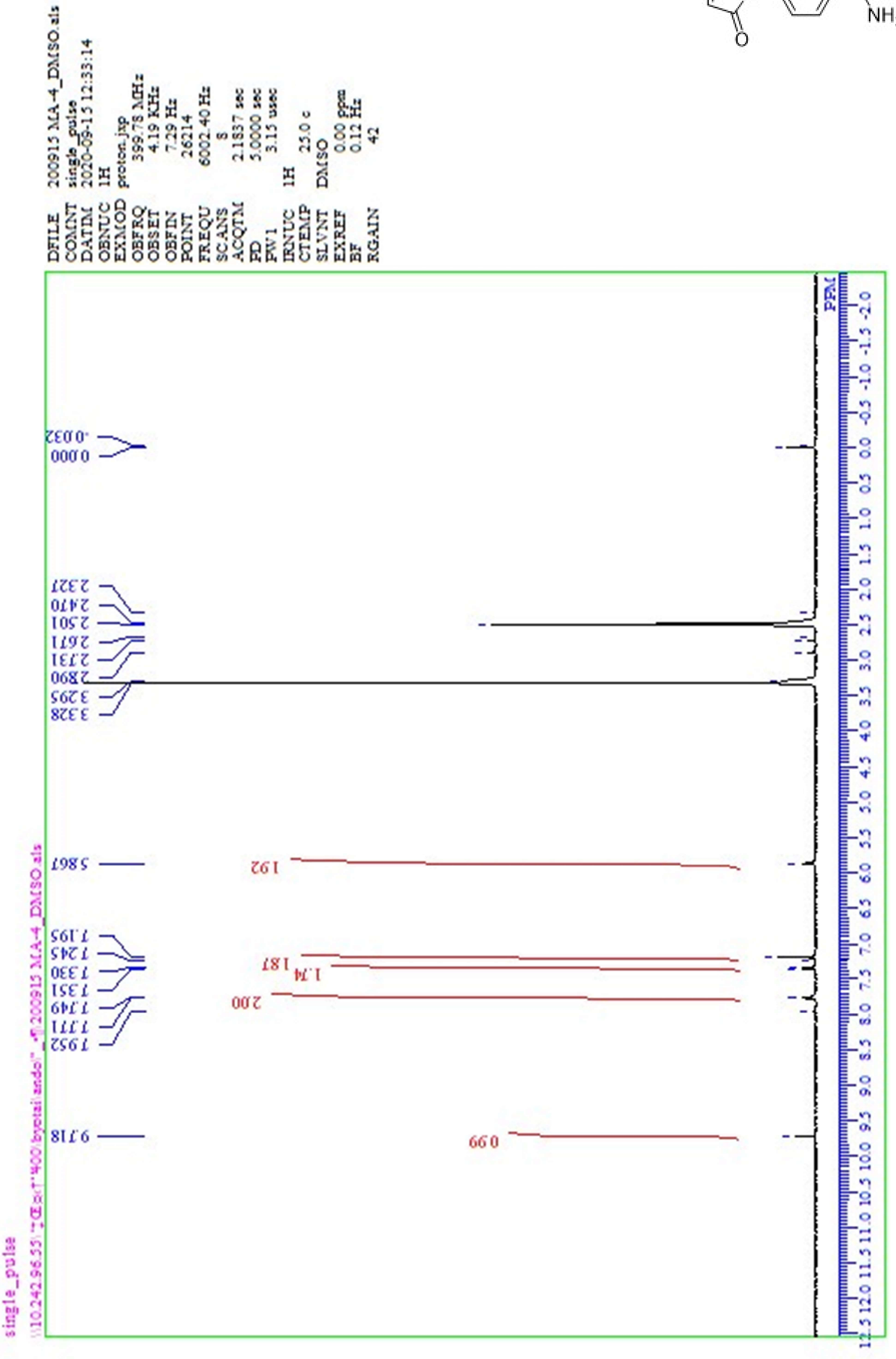

Ham-Mal (Compound **4)** (^{13}C-NMR)

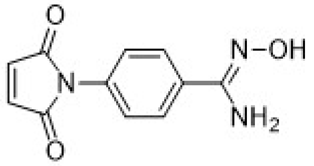

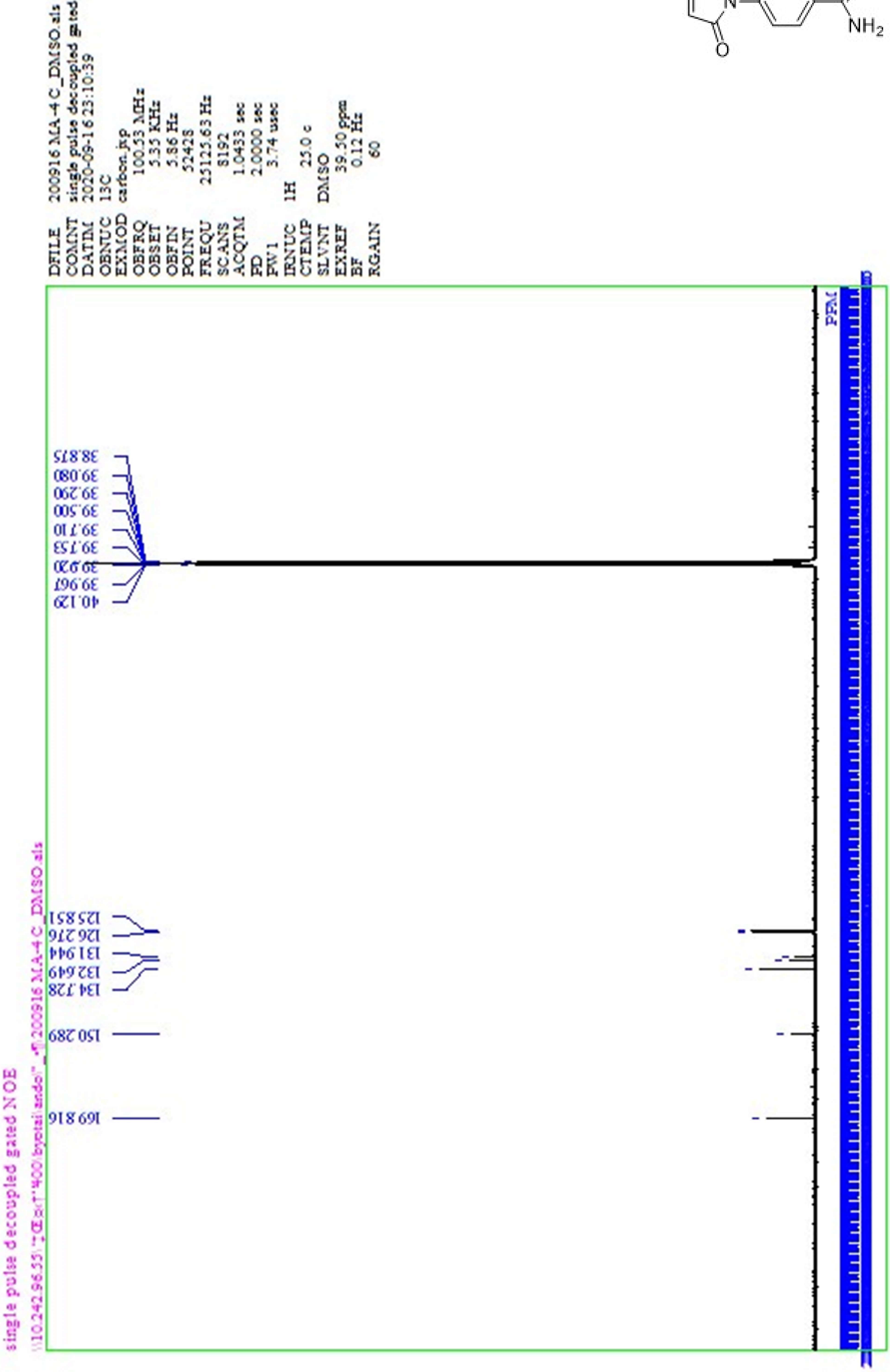

Ham-Cys (Compound **5)** (^{1}H-NMR)

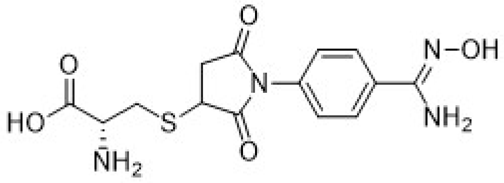

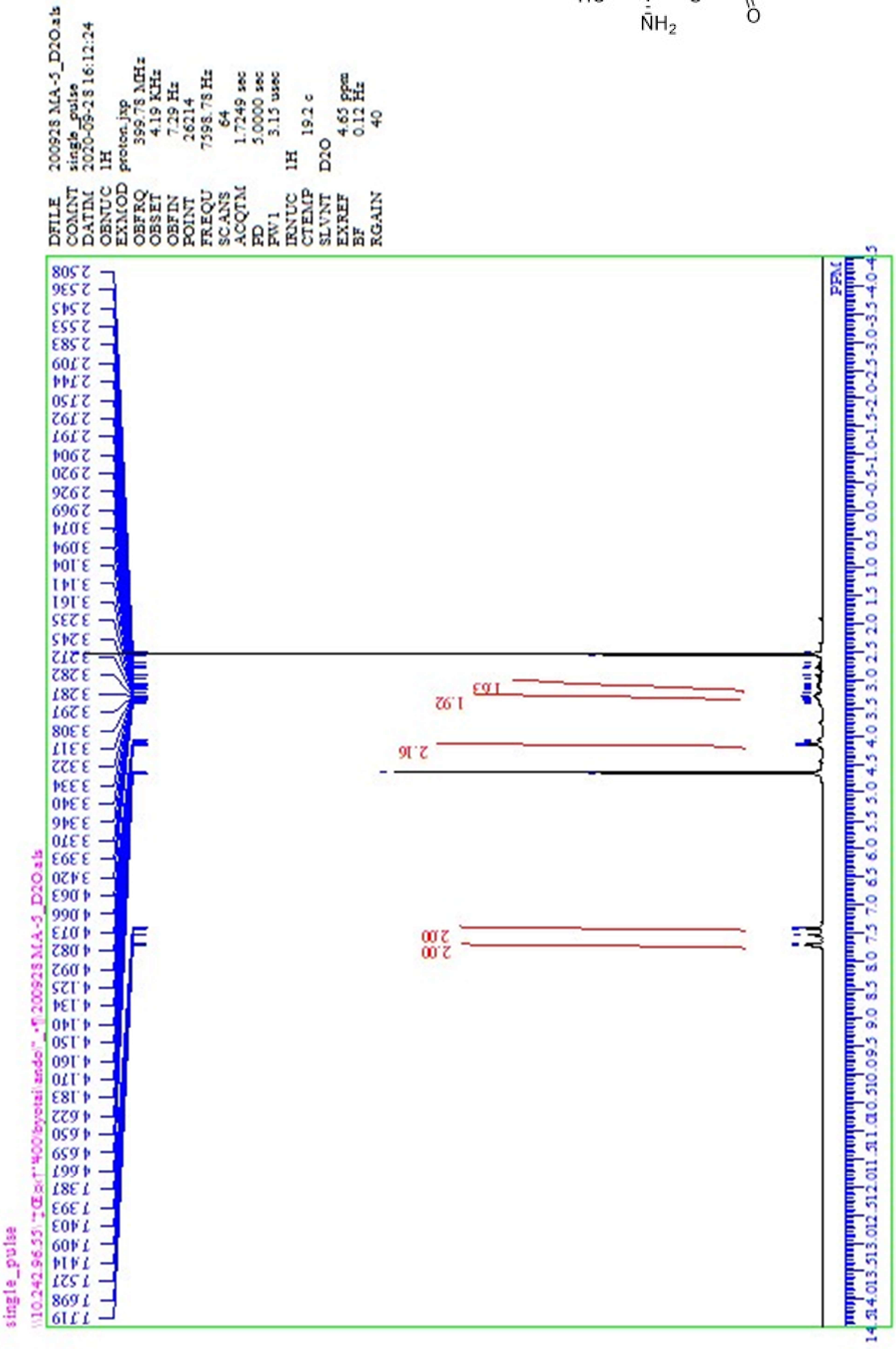

Supplement: Supplementary file 1 — Supplementary Information. [file 41598_2021_98235_MOESM1_ESM.pdf]
